# Supplementary material for: Full-wave electromagnetic modes and hybridization in nanoparticle dimers
Source: Sci Rep. 2019 Oct 10;9:14524. doi: 10.1038/s41598-019-50498-1 (PMC6787262; doi:10.1038/s41598-019-50498-1)
Supplement: Supplementary file 1 — Supplemental Information for Full-wave electromagnetic modes and hybridization in nanoparticle dimers [file 41598_2019_50498_MOESM1_ESM.pdf]

Supplemental Information for "Full-wave electromagnetic modes and hybridization in nanoparticle dimers"

Mariano Pascale[1], Giovanni Miano[1], Roberto Tricarico[1,2], and Carlo Forestiere[1,*]

[1]Department of Electrical Engineering and Information Technology, Università degli Studi di Napoli Federico II, via Claudio 21, Napoli, 80125, Italy
[2]ICFO Institut de Ciències Fotòniques, The Barcelona Institute of Science and Technology, 08860 Castelldefels, Barcelona, Spain
[*]carlo.forestiere@unina.it

ABSTRACT

In this Supplemental Information we first study the scattering properties of an isolated sphere of Silver and Silicon in terms of its resonances and modes. Then, we investigate the scattering response of the Ag homo-dimer studied in the main manuscript, assuming this time the quasi electrostatic approximation. Eventually, we provide additional results for the scattering from Ag and Si homo-dimers, including an complete analysis for a gap-size equal to the spheres'radius.

Scattering from an isolated sphere

n	l	x_{nl}^{TM}	$\omega_{nl}^{\mathrm{TM}}$	ρ_{nl}^{TM}	x_{nl}^{TE}	$\omega_{nl}^{\mathrm{TE}}$	ρ_{nl}^{TE}
1	1	0.8817	3.916	0.6852	-	-	-
1	2	-	-	-	-	-	-
1	3	-	-	-	-	-	-
2	1	1.1744	5.2160	0.1569	-	-	-
2	2	-	-	-	-	-	-
2	3	-	-	-	-	-	-
3	1	1.2124	5.3848	0.1220	-	-	-
3	2	-	-	-	-	-	-
3	3	-	-	-	-	-	-
4	1	1.2382	5.4994	0.1441	-	-	-
4	2	-	-	-	-	-	-
4	3	-	-	-	-	-	-

Table S1. Resonant size parameters x_{nl}^{TM}, x_{nl}^{TE} for TM and TE modes of an isolated $67.5nm$ Ag sphere and corresponding residua.

In this section, we study the scattering properties of an isolated sphere of Silver and Silicon in terms of its resonances and modes. This analysis summarizes the main conclusions of Ref.[1].

First, we calculate the resonances of a silver (Ag) isolated sphere with $R = 67.5$ nm and a silicon (Si) isolated sphere with $R = 100$ nm. We model the Ag permittivity with experimental data[2], while for Si we use a constant permittivity, i.e. $\varepsilon_{R,Si} = 16$. In Tabs. S1 and S2 we list, for few representative modes of the considered Ag and Si isolated sphere, the quantities x_{nl}^{TM} and x_{nl}^{TE} defined as the values of x that minimize the residua introduced in Eq. 5. The resonance frequencies $\omega_{nl}^{\mathrm{TM}}$ and $\omega_{nl}^{\mathrm{TE}}$ can be immediately obtained from x_{nl}^{TM} and x_{nl}^{TE} by using the relation $\omega = cx/R$. We search for the minima in the range $x \in [0.19, 1.80]$ and $x \in [0.01, 100]$ for Ag and Si, respectively. If no minimum is found in the interior of these intervals, we leave the corresponding table cell empty.

In Fig. S1 we show the σ_{sca} spectrum of the Ag nanosphere and the modes responsible of its peaks. The scattering efficiency can be exhaustively described by considering only the fundamental electric dipole and quadrupole modes, namely $\mathbf{e}_{e111}^{\mathrm{TM}}$ and $\mathbf{e}_{e121}^{\mathrm{TM}}$. The peak associated to the mode $\mathbf{e}_{e111}^{\mathrm{TM}}$ is wider compared to $\mathbf{e}_{e121}^{\mathrm{TM}}$ since $\rho_{11}^{\mathrm{TM}} \geq \rho_{21}^{\mathrm{TM}}$. Moreover, there are no asymmetries

n	l	x_{nl}^{TM}	ω_{nl}^{TM}	ρ_{nl}^{TM}	x_{nl}^{TE}	ω_{nl}^{TE}	ρ_{nl}^{TE}
1	1	2.1973	6.5874	1.0368	0.7519	2.2542	0.0722
1	2	1.0561	3.1661	0.1445	1.5478	4.6402	0.0644
1	3	1.9344	5.7992	0.0900	2.3418	7.0206	0.0504
2	1	2.99	8.9639	1.0239	1.0903	3.2687	0.0180
2	2	1.3772	4.1288	0.0206	1.8938	5.6775	0.0301
2	3	2.2207	6.6576	0.0888	2.6884	8.0597	0.0331
3	1	3.8573	11.56	1.0061	1.4217	4.2622	0.0046
3	2	1.703	5.105	0.0035	2.2444	6.7286	0.0131
3	3	2.5225	7.562	0.0314	3.0537	9.1549	0.0217
4	1	4.8458	14.527	0.9829	1.72	5.1565	0.0018
4	2	1.9979	5.9896	0.0114	2.5587	7.6709	0.0134
4	3	2.8477	8.5373	0.0094	3.3762	1.0122	0.0105

Table S2. Resonant size parameters x_{nl}^{TM}, x_{nl}^{TE} for TM and TE modes of an isolated Si sphere and corresponding residua.

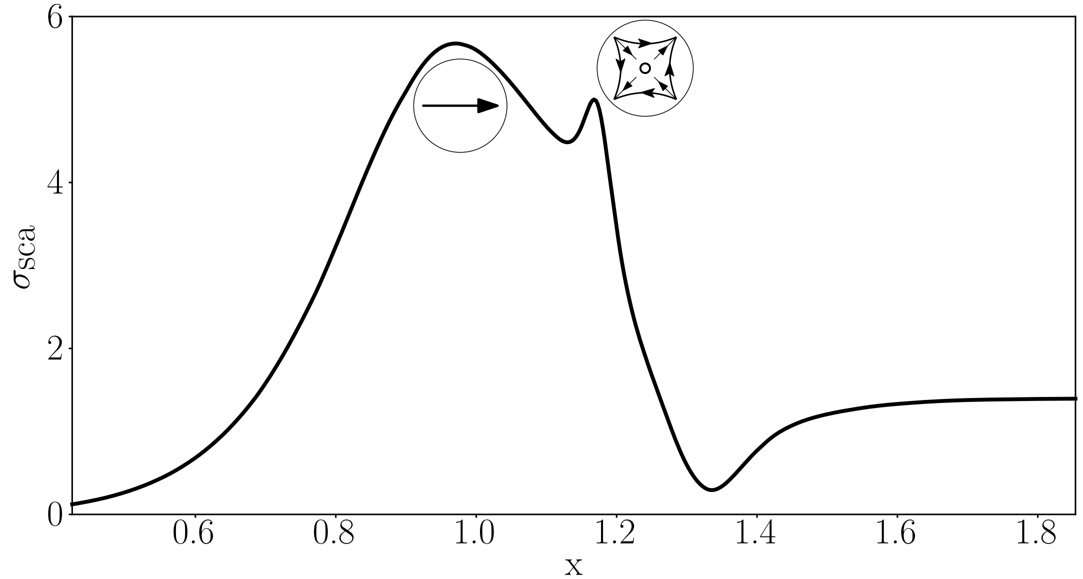

Figure S1. Scattering efficiency of an isolated 67.5*nm* Ag-sphere as a function of the size parameter $x = 2\pi R/\lambda$. The sphere is excited by a linearly polarized plane wave. In the inset the stylized representations of the dominant modes are also shown.

in the spectrum, due to the orthogonality of the fundamental electric modes[1]. In conclusion, the scattering efficiency of an Ag nanoparticle can be exhaustively described by the longitudinal modes of the isolated-sphere.

In Fig. S2 we show the σ_{sca} spectrum of the isolated Si sphere. In the insets we also show the modes responsible of its corresponding peaks. The first peak from the left of σ_{sca} is due to the dominant contribution of the fundamental magnetic dipole $\mathbf{e}_{o111}^{TE}$. The second peak, occurring at $x = 1.02$, originates from the constructive interference between the fundamental electric dipole $\mathbf{e}_{e111}^{TM}$ and the second order electric dipole $\mathbf{e}_{e112}^{TM}$. The third σ_{sca} peak is given by the fundamental magnetic quadrupole $\mathbf{e}_{o121}^{TE}$. The fourth peak is due to second order electric quadrupole $\mathbf{e}_{e122}^{TM}$, and the fifth one to the fundamental magnetic octupole $\mathbf{e}_{o131}^{TE}$. The scattering dip, enclosed by peaks 2 and 3 at $x = 1.076$, originates from the destructive interference between the *broad* fundamental electric dipole $\mathbf{e}_{e111}^{TM}$ and the *narrow* second order electric dipole $\mathbf{e}_{e112}^{TM}$. In conclusion, the scattering efficiency of a Si nanoparticle can be exhaustively described by the transverse modes and the fundamental electric dipole.

Dimer Eigenvalues in the Long-Wavelength Limit

In this section we show the eigenvalues of a homo-dimer with radius R and edge-edge separation $R/4$ in the long-wavelength limit, namely when $k_0 R \ll 1$. As already anticipated in the main manuscript in this limit, the modes $\mathbf{d}_{pmq}^{\|}$ are irrotational everywhere and their eigenvalues $\varepsilon_{pmq}^{\|}$ are negative, size independent, real numbers. Their values for the investigated dimer are shown in Tab. S3. On the contrary, the modes $\mathbf{d}_{pmq}^{\perp}$ are solenoidal everywhere and the quantities $x^2\varepsilon_{pmq}^{\perp}$ are positive, size

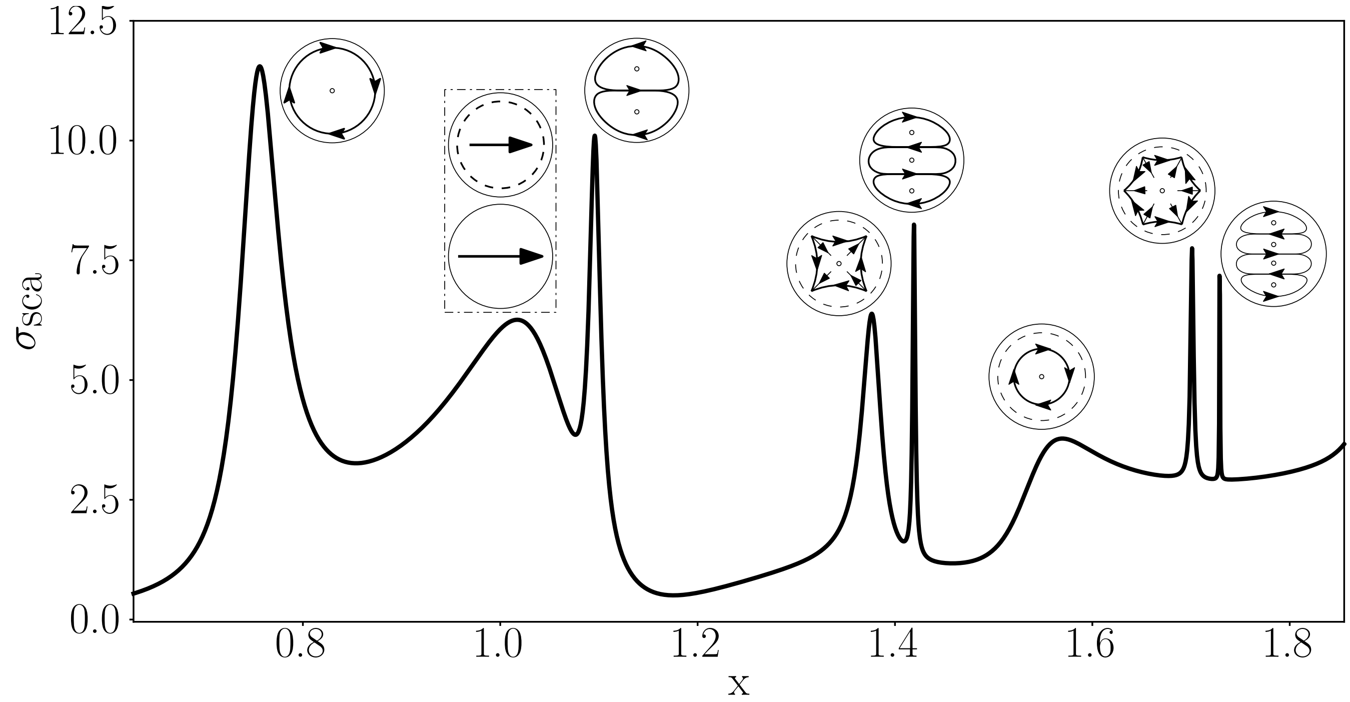

Figure S2. Scattering efficiency of a Si sphere as a function of the size parameter $x = 2\pi R/\lambda$. The sphere is excited by a linearly polarized plane wave. A stylized representation of the modes that dominates the different peaks is shown.

independent real numbers in the long wavelength limit. Their values for the investigated case are shown in Tab. S4- S5.

m	q=1	q=2	q=3	q=4	q=5	q=6
0	-3.16	-1.82	-1.73	-1.47	-1.37	-1.32
1	-2.48	-1.83	-1.75	-1.45	-1.40	-1.31
2	-1.57	-1.47	-1.41	-1.30	-1.30	-1.23
3	-1.34	-1.33	-1.27	-1.24	-1.22	-1.19

Table S3. Eigenvalues $\varepsilon^{\parallel}_{emq}$ in the long-wavelength limit.

m	q=1	q=2	q=3	q=4	q=5	q=6	q=7	q=8
0	9.35	10.40	19.53	20.19	20.19	20.92	32.52	33.22
1	9.61	10.13	19.74	20.19	20.19	20.66	32.69	33.22
2	20.07	20.31	33.00	33.22	33.22	33.43	48.56	48.83
3	33.18	33.25	48.75	48.83	48.83	48.91	66.84	66.95

Table S4. Eigenvalues $\left(x^2 \varepsilon^{\perp}_{emq}\right)$ in the long wavelength limit of a homo-dimer with an edge-edge distance $R/4$ and $q = 1, \dots, 8$.

m	q=9	q=10	q=11	q=12	q=13	q=14	q=15
0	33.22	33.95	39.02	40.09	48.18	48.83	48.83
1	33.22	33.76	39.24	39.77	48.30	48.83	48.83
2	48.83	49.10	59.57	59.80	66.66	66.95	66.95
3	66.95	67.06	82.68	82.76	87.40	87.53	87.53

Table S5. Eigenvalues $\left(x^2 \varepsilon^{\perp}_{emq}\right)$ in the long wavelength limit of a homo-dimer with an edge-edge distance $R/4$ and $q = 9, \dots, 15$.

Scattering from an Ag dimer in the quasi electrostatic approximation

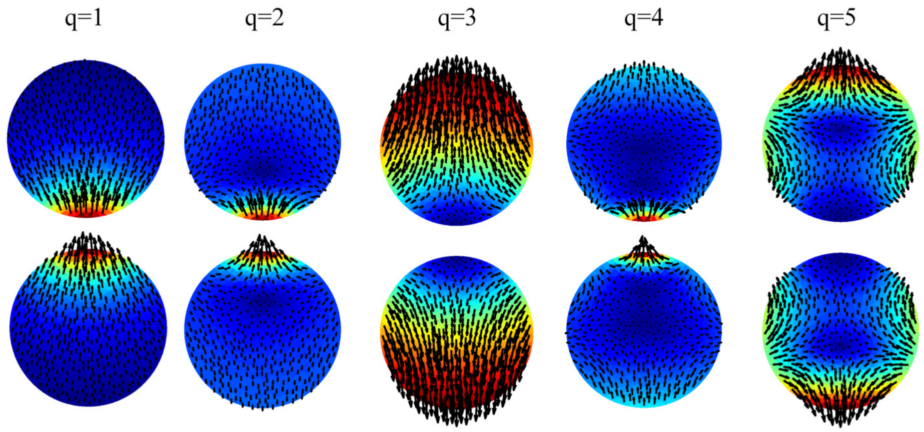

Figure S3. Projection on the $y = 0$ plane of the modes $\mathbf{d}^{||}_{e0q}$ for $q = 1, \ldots 5$. The corresponding eigen-permittivities are shown in Tab. S3. The dimer-modes are bright for $q = 1, 2, 4$.

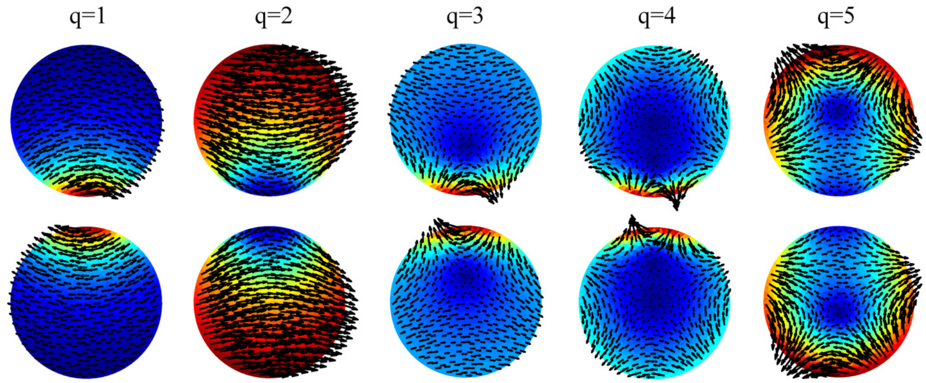

Figure S4. Projection on the $y = 0$ plane of the modes $\mathbf{d}^{||}_{e1q}$ for $q = 1, \ldots 5$. The corresponding eigen-permittivities are shown in Tab. S3. The dimer-modes are bright for $q = 2, 5$.

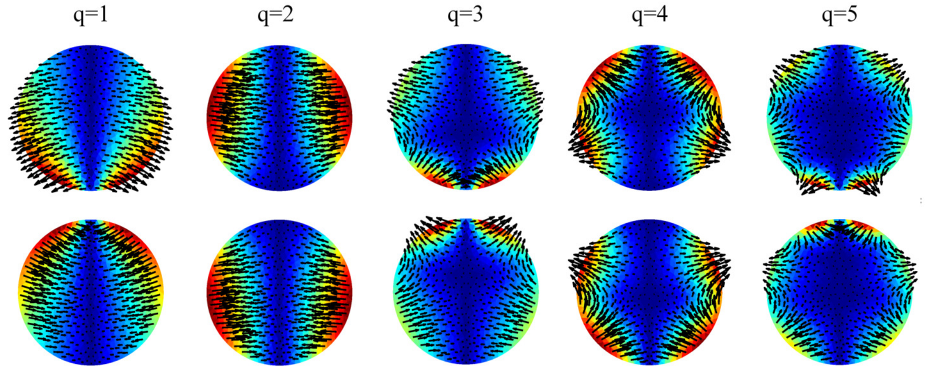

Figure S5. Projection on the $y = 0$ plane of the modes $\mathbf{d}^{||}_{e2q}$ for $q = 1, \ldots 5$. The corresponding eigen-permittivities are shown in Tab. S3.

In this section, we investigate the scattering response of the Ag homo-dimer studied in the main manuscript, assuming this time the quasi electrostatic (Q-ES) approximation. We use the formulation proposed in Ref.[3]. The radius of each sphere

is R and the edge-edge distance is $R/4$. It is worth to remember that in the Q-ES regime only the bright modes, i.e. modes that have a non-vanishing total dipole moment, contribute to the scattering efficiency. In Tab. S3 we list the Q-ES dimer eigen-permittivities, while in Figs. S3, S4, and S5 we show the projections on the $y = 0$ plane of $\mathbf{d}^{\|}_{e0q}$, $\mathbf{d}^{\|}_{e1q}$, and $\mathbf{d}^{\|}_{e2q}$ for $q = 1, \dots, 5$.

Let us consider the scenario in which the dimer is excited by an electric field polarized along the dimer axis (z-axis). In Fig. S6 we show the corresponding scattering efficiency. The two σ_{sca} peaks are caused by the modes $\mathbf{d}^{\|}_{e0,1}$ and $\mathbf{d}^{\|}_{e0,2}$. These modes exhibit non-vanishing total dipole moments along the z-axis. Their eigen-permittivities are $\varepsilon^{\|}_{e0,1} = -3.16$ and $\varepsilon^{\|}_{e0,2} = -1.82$. The corresponding resonant frequencies are $\omega^{\|}_{e0,1} = 5.01$ Prad/s and $\omega^{\|}_{e0,2} = 5.36$ Prad/s while the resonant size parameters are $x^{\|}_{e0,1} = 1.13$ and $x^{\|}_{e0,2} = 1.21$, which are highlighted in Fig. S6 with vertical dashed lines.

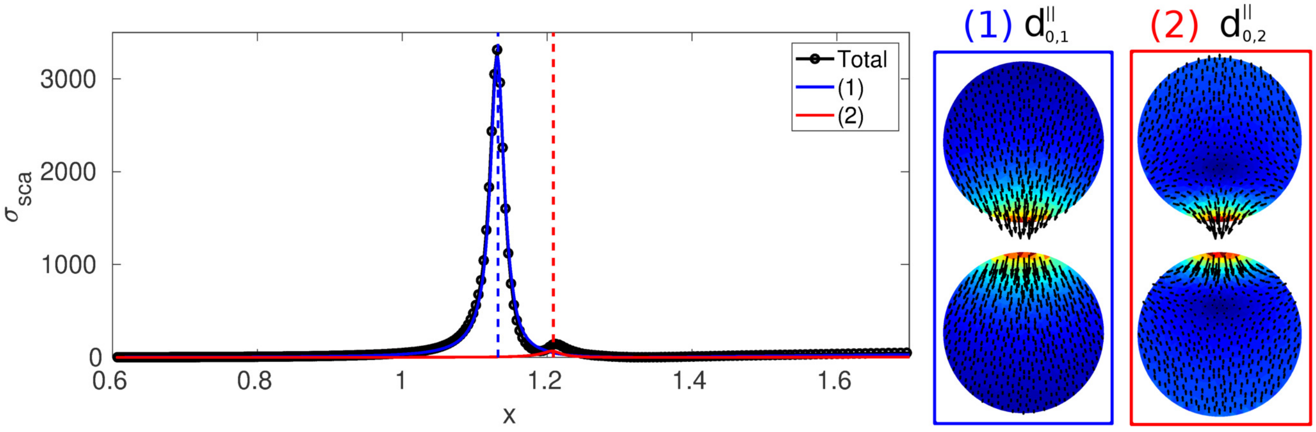

Figure S6. Scattering efficiency σ_{sca} of an Ag-spheres homo-dimer as a function of the spheres size parameter $x = 2\pi R/\lambda$, obtained under the Q-ES approximation. The radius of each sphere is R, the edge-edge distance is $R/4$. The dimer is excited by an electric field polarized parallel to the dimer's axis. Partial scattering cross section (in color) of the two dominant dimer-modes whose projections on the $y = 0$ plane are shown on the right.

In Fig. S7 we show the scattering efficiency of the Ag dimer when it is excited by an electric field polarized transversely with respect to the dimer axis. The peak of σ_{sca} is due to the mode $\mathbf{d}^{\|}_{e1,2}$. The mode $\mathbf{d}^{\|}_{e1,4}$ gives a very small contribution. Their eigen-permittivities are $\varepsilon^{\|}_{e1,2} = -1.83$, $\varepsilon^{\|}_{e1,4} = -1.40$. The the corresponding resonant frequencies are 5.35 Prad/s and 5.46 Prad/s and the corresponding resonant size parameters are $x^{\|}_{e1,2} = 1.20$ and $x^{\|}_{e1,2} = 1.23$, which are highlighted in Fig. S7 with vertical dashed lines.

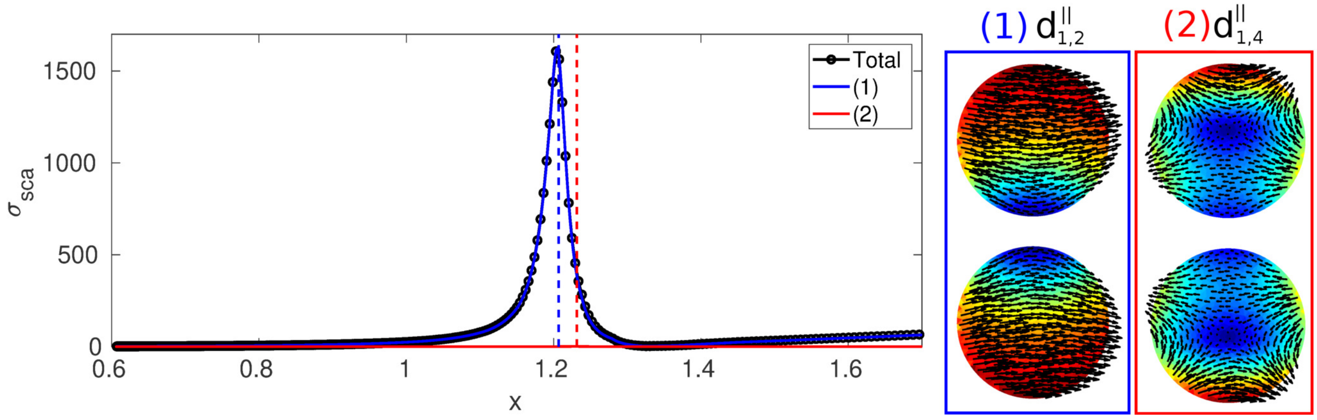

Figure S7. Scattering efficiency σ_{sca} of an Ag-spheres homo-dimer as a function of the spheres size parameter $x = 2\pi R/\lambda$, obtained under the Q-ES approximation. The radius of each sphere is R, the edge-edge distance is $R/4$. The dimer is excited by an electric field polarized orthogonally to the dimer axis. Partial scattering cross section (in color) of the two dominant dimer-modes whose projections on the $y = 0$ plane are shown on the right.

Additional results on the homo-dimer with gap $R/4$

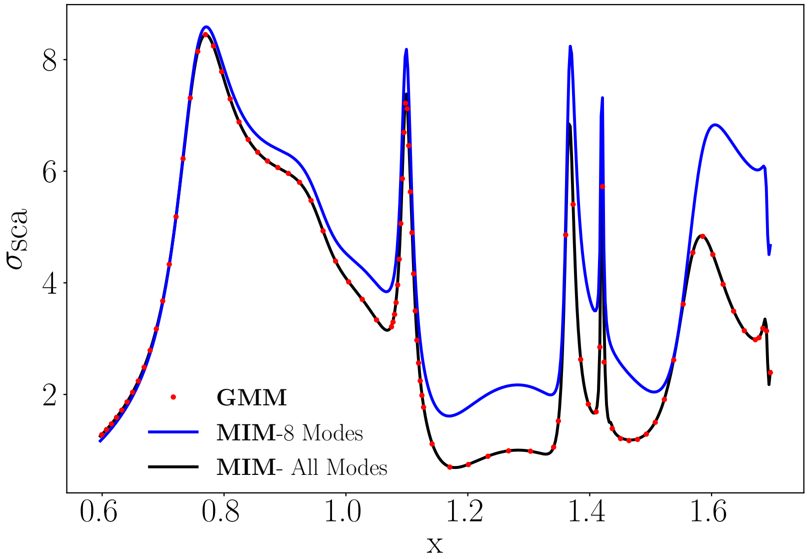

Figure S8. Scattering efficiency σ_{sca} of a Si-spheres homo-dimer as a function of the size parameter $x = 2\pi R/\lambda$, obtained via the material-independent-mode expansion considering all the dimer-modes (black line), only the eight dimer-modes shown on the right of Fig. 10 (blue line), and by the GMM direct-calculation (red dots). The radius of each sphere is R, the edge-edge distance $R/4$. The dimer is excited by a plane wave polarized along the dimer axis and propagating orthogonally to it.

In Fig. S8 we plot the scattering efficiency σ_{sca} of a Si homo-dimer with an edge-edge separation $R/4$. The homo-dimer is excited by an incident field that is polarized along the dimer's axis $\hat{\mathbf{z}}$, while it is propagating along the transverse direction $\hat{\mathbf{x}}$. In particular, we calculate the scattering efficiency σ_{sca} by using the material-independent-mode expansion considering all the dimer-modes (black line), only the eight dimer-modes shown on the right of Fig. 10, and by the direct GMM calculation (red dots) as a function of the size parameter x. We note that we obtain a satisfactory agreement by only using eight modes in the expansion.

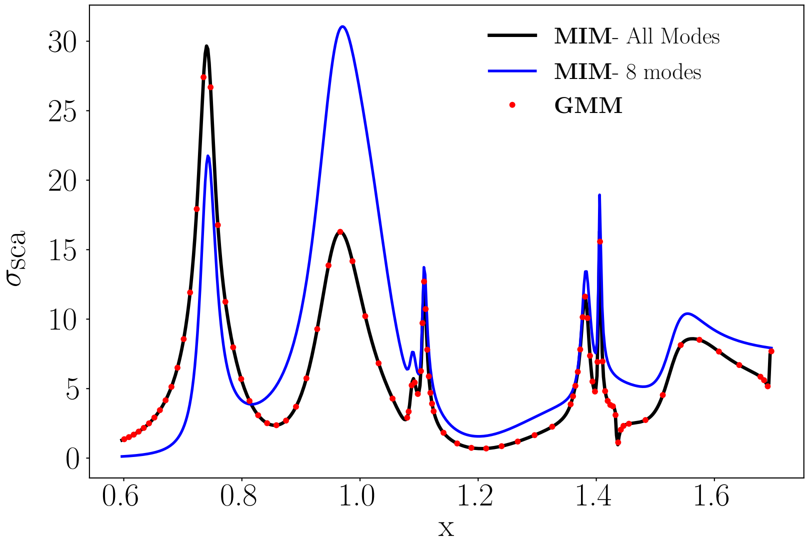

Figure S9. Scattering efficiency σ_{sca} of a Si-spheres homo-dimer as a function of the spheres size parameter $x = 2\pi R/\lambda$, obtained via the material-independent-mode expansion considering all the dimer-modes (black line), only the eight dimer-modes shown on the right of Fig. 16 (blue line), and by the GMM direct-calculation (red dots). The radius of each sphere is R, the edge-edge distance $R/4$. The dimer is excited by a plane wave propagating along the dimer's axis and polarized orthogonally to it.

In Fig. S9 we plot the scattering efficiency σ_{sca} of a Si homo-dimer with an edge-edge separation $R/4$. The homo-dimer is excited by an incident field that is propagating along the dimer's axis $\hat{\mathbf{z}}$, while it is polarized along the transverse direction $\hat{\mathbf{x}}$. In particular, we calculate the scattering efficiency σ_{sca} by using the material-independent-mode expansion considering all the dimer-modes (black line), only the eight dimer-modes shown on the right of Fig. 16, and by the direct GMM calculation (red dots) as a function of the size parameter x. We note that we obtain a satisfactory agreement by only using eight modes in the expansion.

Next, we investigate the spatial distribution of the total electric field within the dimer in correspondence of the peaks of the scattering efficiency for the scenarios investigated in the main manuscript.

First, we consider the Ag homo-dimer with $R = 67.5nm$ and an edge-edge distance $R/4$. In Fig. S10 we show the projection of real part of the scattered electric field on the plane $y = 0$, in correspondence of the two σ_{sca} peaks of Fig. 4 at $x = 0.747$ (left) and at $x = 1.15$ (right). The Ag homo-dimer is excited by a plane wave propagating orthogonally to the dimer and polarized along the dimer axis. It is apparent that the near field distributions at $x = 0.747$ and $x = 1.15$ are dominated by the modes $\mathbf{d}^{\parallel}_{e0,1}$ and $\mathbf{d}^{\parallel}_{e1,3}$, respectively, which also dominate the far-field response.

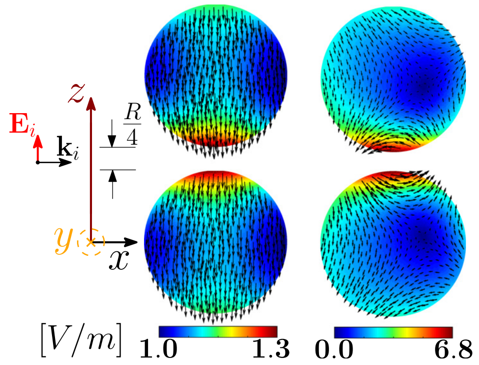

Figure S10. Real part of the projection on the $y = 0$ plane of the scattered electric field at the first and second peak of the scattering spectrum of an Ag homo-dimer (shown in Fig. 4) when it is excited by a plane wave polarized along the dimer axis.

In Fig. S11 we show the projection of real part of the scattered electric field on the plane $y = 0$, in correspondence of the σ_{sca} peaks of Fig. 7 at $x = 0.892$ (left) and at $x = 1.183$ (right). The homo-dimer is excited by a plane wave propagating parallel to the dimer axis and polarized orthogonally to it. It is apparent that the near field distribution at $x = 0.892$ consistently differs from the modes $\mathbf{d}^{\parallel}_{e1,1}$ which dominates the far-field response. On the contrary at $x = 1.183$ (right) the near-field distribution closely resembles the mode $\mathbf{d}^{\parallel}_{e1,4}$, which is responsible for the second peak of σ_{sca}.

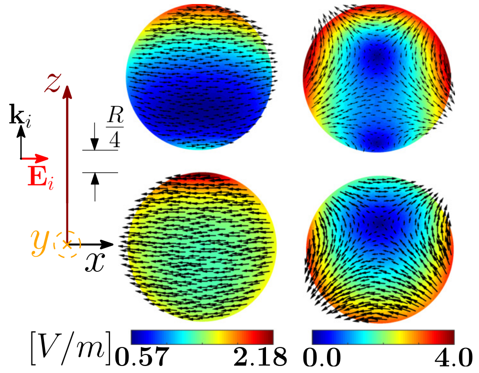

Figure S11. Real part of the projection on the $y = 0$ plane of the scattered electric field at the first and second peak of the scattering spectrum of an Ag homo-dimer (shown in Fig. 7) when it is excited by a plane wave polarized orthogonally to the dimer axis.

We now investigate the Si homo-dimer with $R = 100nm$ and edge-edge distance $R/4$. In Fig. S12 we show the projection of real part of the scattered electric field on the plane $y = 0$ in correspondence of the σ_{sca} peaks of Fig. 10, The homo-dimer is excited by a plane wave propagating orthogonally to the dimer axis and polarized along it. It is apparent from Fig. S12 the effect

of the propagation along the horizontal horizontal direction. Nevertheless, the electric field distributions in correspondence of the peaks only roughly resemble the modes shown in Fig. 10.

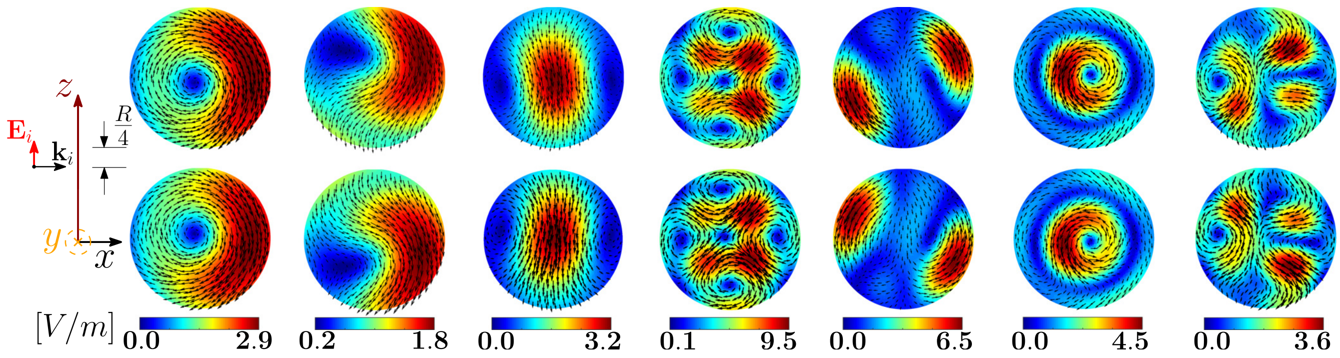

Figure S12. Real part of the projection on the $y = 0$ plane of the scattered electric field at the peaks of the scattering spectrum of a Si homo-dimer (from left to right $x = 0.771$, $x = 0.901$ $x = 1.099$, $x = 1.366$, $x = 1.423$, $x = 1.584$, $x = 1.688$). The homo-dimer is excited by a plane wave polarized along to the dimer axis, and propagating orthogonally to it.

In Fig. S13 we show the projection of real part of the scattered electric field on the plane $y = 0$ at the σ_{sca} peaks of Fig. 16. The homo-dimer is excited by a plane wave propagating orthogonally to the dimer and polarized along the dimer axis. It is apparent from Fig. S13 the effect of the propagation along the vertical z direction. Nevertheless, the electric field distributions in correspondence of the peaks only roughly resemble the modes shown in Fig. 10.

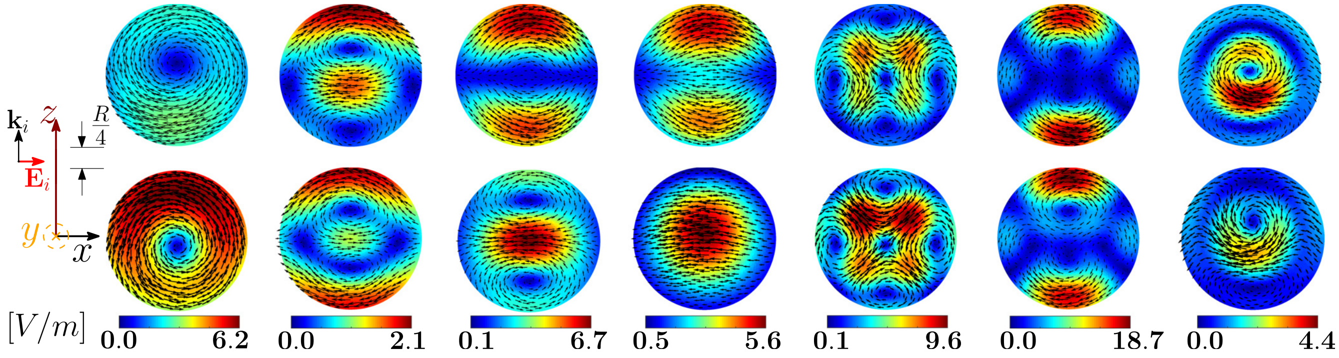

Figure S13. Real part of the projection on the $y = 0$ plane of the scattered electric field distribution at the peaks of the scattering spectrum of an Si homo-dimer (shown in Fig. 16) (from left to right $x = 0.740$, $x = 0.967$, $x = 1.090$, $x = 1.108$, $x = 1.381$, $x = 1.406$, $x = 1.560$) when it is excited by a plane wave polarized orthogonally to the dimer axis.

Scattering Analysis as the gap size varies

In this section, we increase the gap size of the dimer from $R/4$ to R. Although, as we vary the distance between two spheres, the dimer-modes change, we still denote, by an abuse of notation, the modes in this new configuration as $\mathbf{d}^{\parallel}_{pm,q}$ and $\mathbf{d}^{\perp}_{pm,q}$. The sorting of the dimer-modes is induced by the corresponding eigen-permittivities. In the limit $x \to 0$, the eigen-permittivities are real and they are sorted in an ascending order. The same ordering is kept for finite values of x, by following each eigen-permittivity on the complex plane as x increases.

Longitudinally polarized Ag homo-dimer

We study a silver homo-dimer with $R = 67.5nm$, and edge-edge separation $67.5nm$. We consider the modes that are excited by an incident field that is polarized along the dimer's axis $\hat{\mathbf{z}}$, while it is propagating along the transverse direction $\hat{\mathbf{x}}$. In Fig. S14, we plot the scattering efficiency σ_{sca} obtained by using the material-independent-mode (MIM) expansion of Eq. 1 (black line), and by the direct GMM calculation (red dots) as a function of the size parameter x. The two results are in very good agreement. We also show in color the partial scattering efficiencies of three dominant dimer modes, whose real projections on the $y = 0$ plane are represented on the right. The analysis of the partial scattering efficiencies reveals that the dimer mode $\mathbf{d}^{\parallel}_{e0,1}$

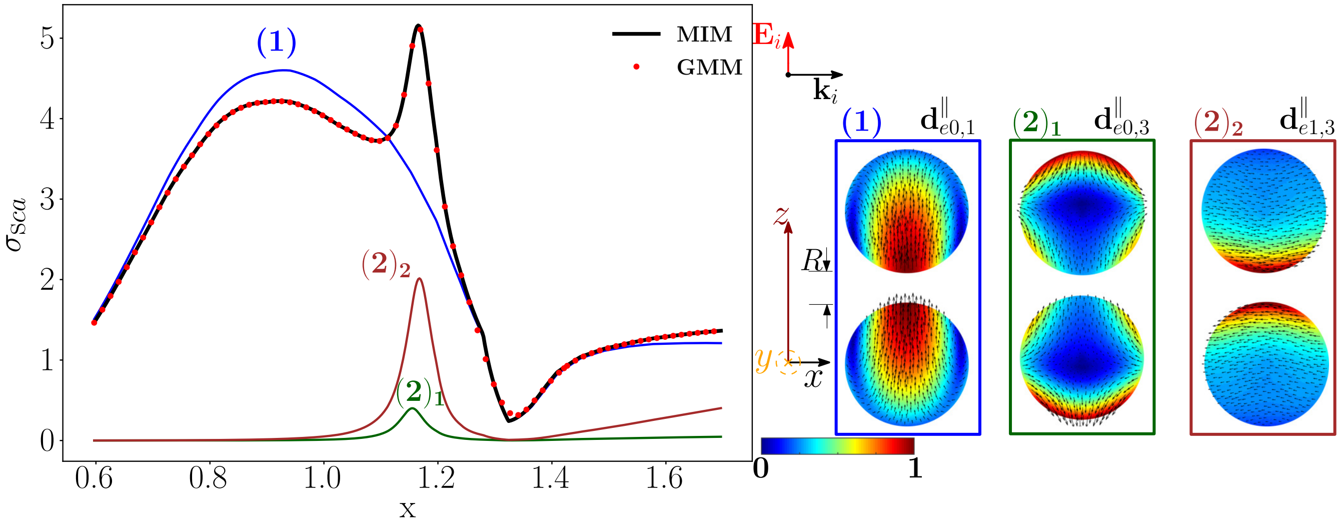

Figure S14. Scattering efficiency σ_{sca} of an Ag homo-dimer as a function of the spheres size parameter $x = 2\pi R/\lambda$, obtained via material-independent-mode expansion (black line) and by direct-calculation (red dots). The radius of each sphere is $R = 67.5$ nm, the edge-edge distance 67.5 nm. The dimer is excited by a plane wave propagating orthogonally to the dimer and polarized along the dimer's axis. Partial scattering efficiencies (in color) of three dominant dimer modes whose xz-plane projections are shown on the right.

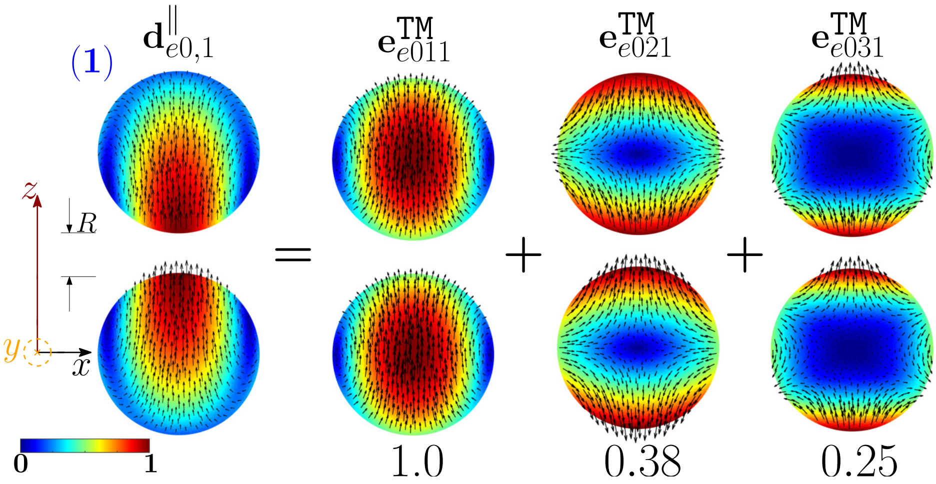

Figure S15. Decomposition of the dimer mode $\mathbf{d}^{\|}_{e0,1}$ at $x = 0.912$, in terms of hybridizing isolated-sphere modes (real part of the projection on the $y = 0$ plane). Each isolated sphere mode is multiplied by the corresponding expansion coefficient of Eq. 8. Below each isolated-sphere mode its hybridization weight $H^{\|\,|\mathrm{TM}}_{e01\,nl}$ is shown. The NPs distance is not to scale.

dominates the total scattering efficiency at its first peak. The mode $\mathbf{d}^{\|}_{e0,1}$ originates from the hybridization of the isolated-sphere modes shown in Fig. S15 together with the corresponding hybridization weights. Specifically, the fundamental electric dipole $\mathbf{e}^{\mathrm{TM}}_{e011}$, quadrupole $\mathbf{e}^{\mathrm{TM}}_{e021}$, and octupole $\mathbf{e}^{\mathrm{TM}}_{e031}$ interfere constructively in the proximity of the dimer gap. The same modes interfere destructively in the regions located diametrically opposite to the gap.

We examine the second peak of the scattering efficiency. It is dominated by the mode $\mathbf{d}^{\|}_{e1,3}$. Now, we analyse in detail its decomposition in terms of isolated-sphere modes with the help of Fig. S16. The fundamental electric dipole $\mathbf{e}^{\mathrm{TM}}_{e111}$, quadrupole $\mathbf{e}^{\mathrm{TM}}_{e121}$ and the fundamental magnetic dipole $\mathbf{e}^{\mathrm{TE}}_{o111}$ interfere constructively in the proximity of the gap, while they undergo destructive interference in the regions of the two spheres diametrically opposite to the gap. The second and third order electric dipole interfere destructively in the neighbourhood of the sphere's center.

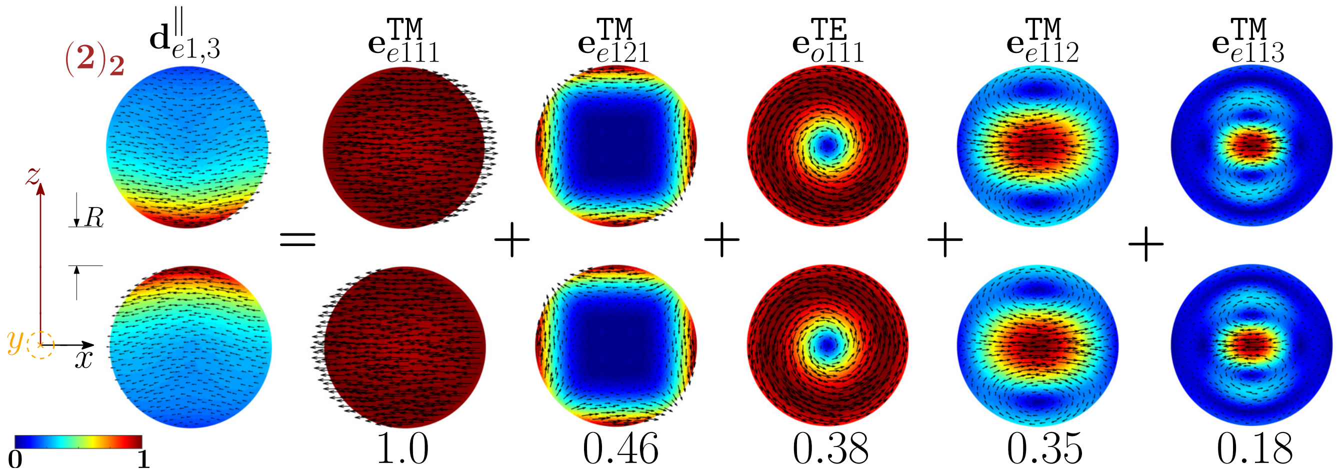

Figure S16. Decomposition of the dimer mode $\mathbf{d}^{\parallel}_{e1,3}$ at $x = 1.16$, in terms of hybridizing isolated-sphere modes (real part of the projection on the $y = 0$ plane). Each isolated sphere mode is multiplied by the corresponding expansion coefficient of Eq. 8. Below each isolated-sphere mode its hybridization weight $H^{\parallel|\mathrm{TM}}_{e13nl}$ ($H^{\parallel|\mathrm{TE}}_{o13nl}$) is shown. The NPs distance is not to scale.

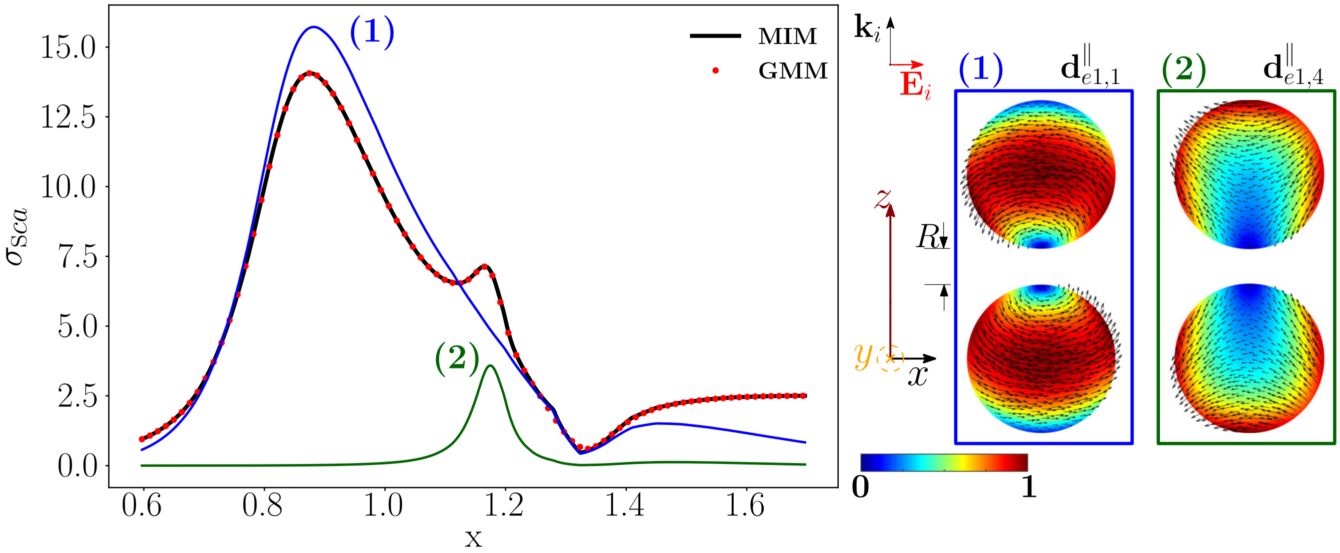

Figure S17. Scattering efficiency σ_{sca} of an Ag-spheres homo-dimer as a function of the spheres size parameter $x = 2\pi R/\lambda$, obtained via the material-independent-mode expansion (black line) and by direct-calculation[4] (red dots). The radius of each sphere is $R = 67.5$ nm, the edge-edge distance is 67.5 nm. The dimer is excited by a plane wave propagating along the dimer's axis and polarized orthogonally to it. Partial scattering cross section (in color) of two dominant dimer modes whose real part projections on the $y = 0$ plane are shown on the right.

Transversely polarized Ag homo-dimer

We now consider an identical Ag homo-dimer illuminated by a plane-wave polarized along the $\hat{\mathbf{x}}$-direction, and propagating along the direction $\hat{\mathbf{z}}$. In Fig. S17, we plot the corresponding scattering efficiency obtained from both the mode expansion 1 (black line) and by direct GMM calculation (red dots). We also show in color the partial scattering efficiency of the dimer modes $\mathbf{d}^{\parallel}_{e1,1}$, $\mathbf{d}^{\parallel}_{e1,4}$ dominating the scattering response. Their projections on the $y = 0$ plane (real part) are shown on the right.

The dimer mode $\mathbf{d}^{\parallel}_{e1,1}$, responsible for the first σ_{sca} peak originates from the hybridization of the isolated-sphere modes shown in Fig. S18. In particular, the fundamental electric dipole $\mathbf{e}^{\mathrm{TM}}_{e111}$ and quadrupole $\mathbf{e}^{\mathrm{TM}}_{e121}$ and magnetic dipole $\mathbf{e}^{\mathrm{TE}}_{o111}$ interfere destructively in the close proximity of the gap, where the mode reaches a minimum.

The dimer mode $\mathbf{d}^{\parallel}_{e1,4}$ arises from the interaction of the isolated-sphere modes shown in Fig. S19. The fundamental electric quadrupole $\mathbf{e}^{\mathrm{TM}}_{e121}$ and octupole $\mathbf{e}^{\mathrm{TM}}_{e131}$ interfere destructively in the proximity of the gap. The fundamental electric quadrupole

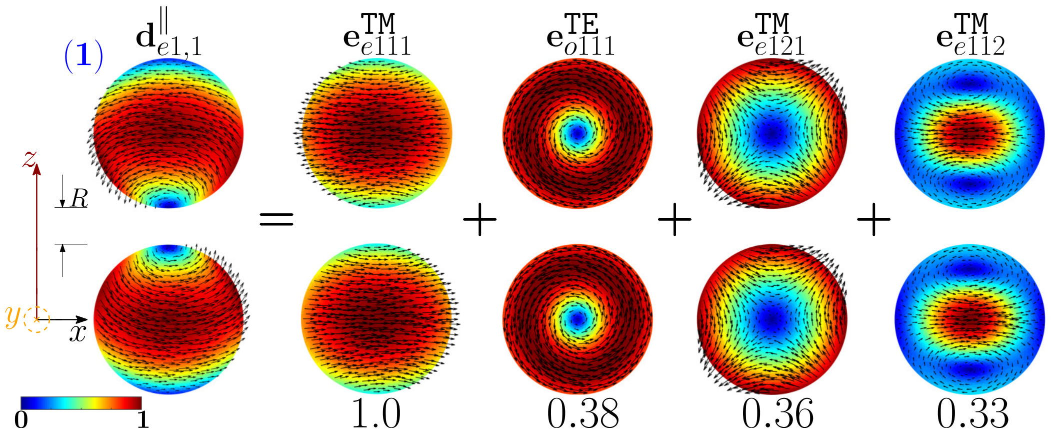

Figure S18. Decomposition of the dimer mode $\mathbf{d}^{\parallel}_{e1,1}$ at $x = 0.874$, in terms of hybridizing isolated-sphere modes (real part of the projection on the $y = 0$ plane). Each isolated sphere mode is multiplied by the corresponding expansion coefficient of Eq. 8. Below each isolated-sphere mode its hybridization weight $H^{\parallel|\mathtt{TM}}_{e11nl}$ ($H^{\parallel|\mathtt{TE}}_{o11nl}$) is shown. The NPs distance is not to scale.

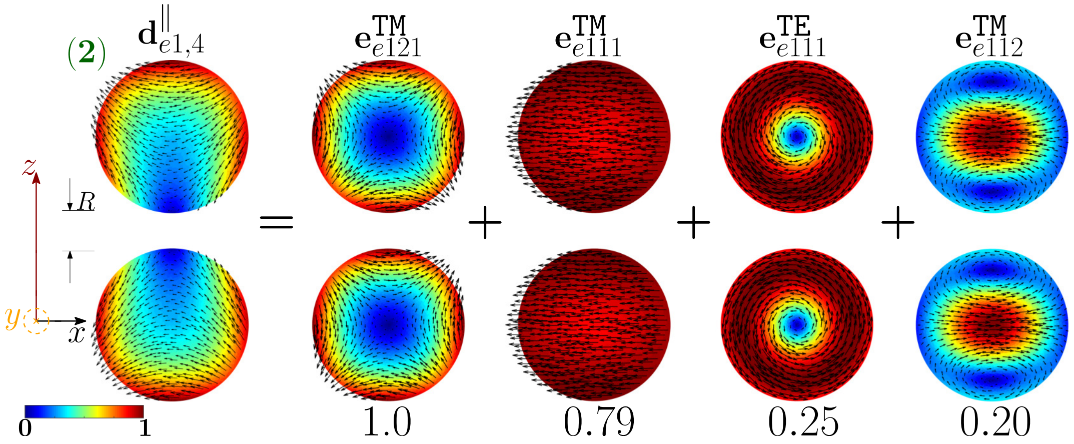

Figure S19. Decomposition of the dimer mode $\mathbf{d}^{\parallel}_{e1,4}$ at $x = 1.165$, in terms of hybridizing isolated-sphere modes (real part of the projection on the $y = 0$ plane). Each isolated sphere mode is multiplied by the corresponding expansion coefficient of Eq. 8. Below each isolated-sphere mode its hybridization weight $H^{\parallel|\mathtt{TM}}_{e14nl}$ ($H^{\parallel|\mathtt{TE}}_{o14nl}$) is shown. The NPs distance is not to scale.

and the electric dipole also interfere destructively in this region. The modes $\mathbf{e}^{\mathrm{TM}}_{e121}$ and $\mathbf{e}^{\mathrm{TM}}_{e131}$ interfere constructively on the region of each sphere opposite to the gap.

Longitudinally polarized Si homo-dimer

Now, we investigate the scattering from a homo-dimer of the same geometry but made of a dielectric material, i.e. Silicon, with permittivity $\varepsilon_R = 16$. The incident plane wave is polarized along the dimer axis $\hat{\mathbf{z}}$, while it is propagating along the transverse direction $\hat{\mathbf{x}}$. In Fig. S20, we plot the scattering efficiency obtained by the material-independent-mode expansion 1 (black line) and by direct GMM calculation[4] (red dots) as a function of the size parameter x. We also show in color the partial scattering efficiency of the seven dominant dimer modes, whose real projections on the $y = 0$ plane are represented on the right.

As shown in Fig. S21, the dimer-mode $\mathbf{d}^{\perp}_{e1,2}(\mathbf{r})$, which is responsible for the first peak of σ_{sca}, arise from the hybridization of the fundamental magnetic dipole $\mathbf{e}^{\mathrm{TE}}_{o111}$, and the first and second order electric dipoles, i.e. $\mathbf{e}^{\mathrm{TM}}_{e111}$, $\mathbf{e}^{\mathrm{TM}}_{e112}$. The modes $\mathbf{e}^{\mathrm{TM}}_{e111}$ and $\mathbf{e}^{\mathrm{TM}}_{e112}$ constructively interfere with $\mathbf{e}^{\mathrm{TE}}_{o111}$ within the two hemispheres located closer to the gap and destructively in the remaining half-spheres. The net effect is to move the vortex core away from the gap in each sphere.

The second σ_{sca} peak arise from the interference between the dimer-modes $\mathbf{d}^{\parallel}_{e0,1}(\mathbf{r})$ and $\mathbf{d}^{\perp}_{e0,3}(\mathbf{r})$. The third peak is mainly due to the mode $\mathbf{d}^{\perp}_{e2,1}(\mathbf{r})$. In Fig. S22 we show the dimer mode behind the fourth σ_{sca} peak, namely $\mathbf{d}^{\perp}_{e1,7}$. It arises from the hybridization among the second order electric quadrupole $\mathbf{e}^{\mathrm{TM}}_{e122}$, which dominates the hybridization, and the second order magnetic dipole $\mathbf{e}^{\mathrm{TE}}_{o112}$, the third order electric dipole $\mathbf{e}^{\mathrm{TM}}_{e113}$, and the fundamental magnetic quadrupole $\mathbf{e}^{\mathrm{TE}}_{o121}$. We recall that for

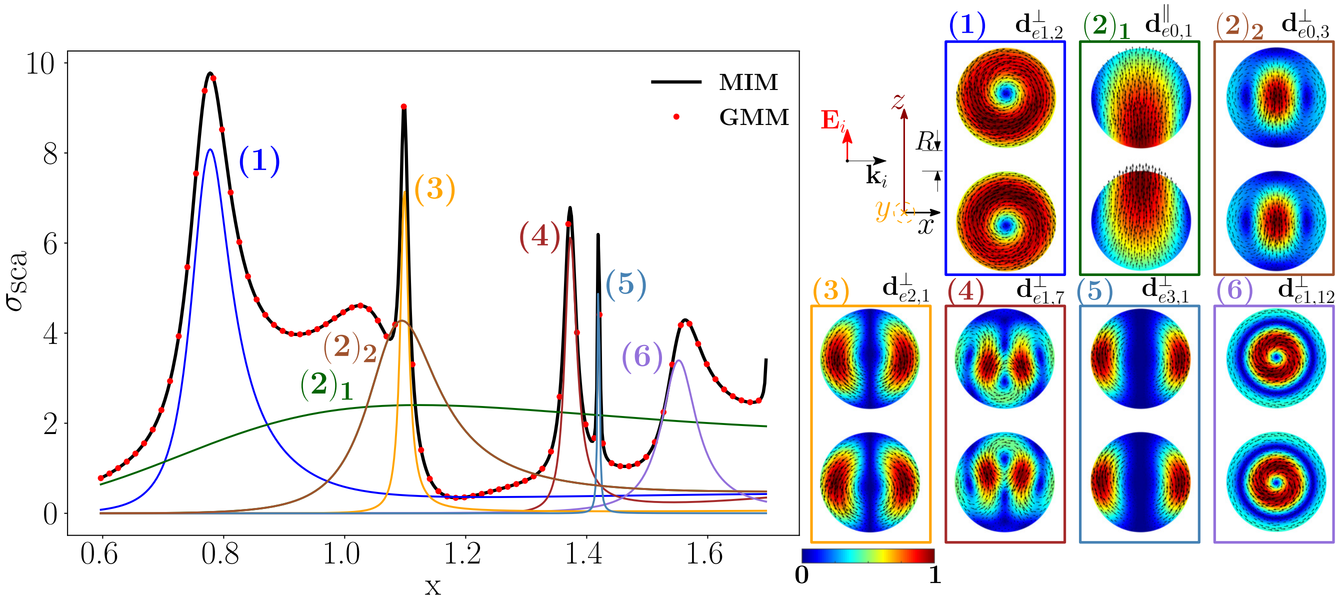

Figure S20. Scattering efficiency σ_{sca} of a Si-spheres homo-dimer as a function of the size parameter $x = 2\pi R/\lambda$, obtained via material-independent-mode expansion (black line) and by direct-calculation (red dots). The radius of each sphere is R, the edge-edge distance R. The dimer is excited by a plane wave propagating orthogonally to the dimer and polarized along the dimer axis. Partial scattering efficiency (in color) of seven dominant dimer modes whose $y = 0$-plane projections are shown on the right.

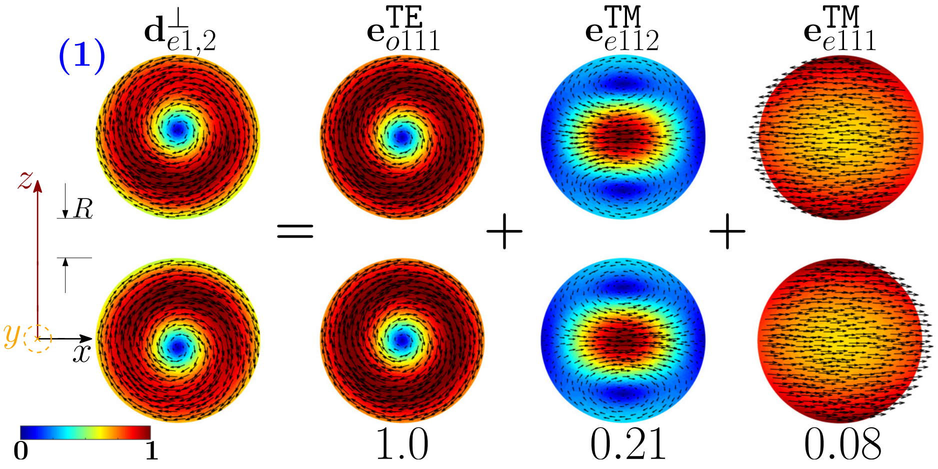

Figure S21. Decomposition of the dimer mode $\mathbf{d}^{\perp}_{e1,2}$ at $x = 0.779$ in terms of hybridizing isolated-sphere modes (real part of the projection on the $y = 0$ plane). Each isolated sphere modes is multiplied by the expansion coefficients of Eq. 8. Below each isolated-sphere mode we also show its hybridization weight $H^{\perp|\text{TM}}_{e12nl}$ ($H^{\perp|\text{TE}}_{o12nl}$). The NPs distance is not to scale.

a gap size of $R/4$, the corresponding dimer-mode obtained shown in Fig. 13 of the main manuscript, the hybridization was dominated by the second order magnetic dipole.

Transversely polarized Si homo-dimer

We now still consider an identical silicon homo-dimer, but under different excitation conditions. The incident plane-wave is polarized along the transverse $\hat{\mathbf{x}}$-direction and propagating along the longitudinal direction $\hat{\mathbf{z}}$. In Fig. S23, we plot the scattering efficiency obtained by the material-independent-mode expansion of Eq. 1 (black line), and by direct GMM calculation[4] (red dots) as a function of the size parameter x.

In Fig. S24 the mode $\mathbf{d}^{\perp}_{e1,2}$, which causes the first peak of the σ_{sca} spectrum, is decomposed in terms of hybridizing isolated-sphere modes. It almost coincides with the isolated-sphere fundamental magnetic dipole apart from a small contribution

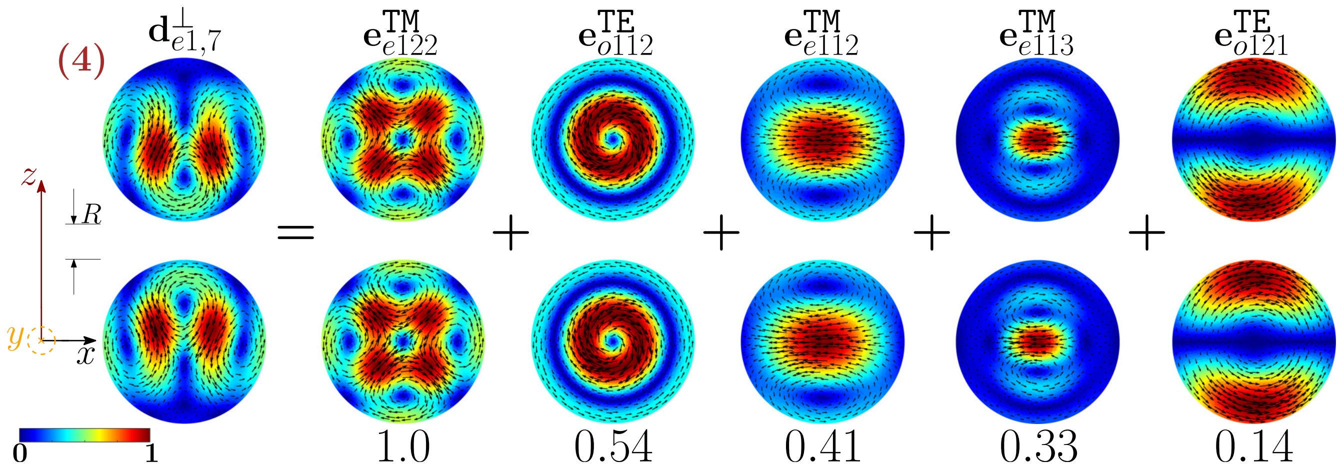

Figure S22. Decomposition of the dimer mode $\mathbf{d}^{\perp}_{e1,7}$ at $x = 1.372$ in terms of hybridizing isolated-sphere modes (real part of the projection on the $y = 0$ plane). Each isolated sphere modes is multiplied by the expansion coefficients of Eq. 8. Below each isolated-sphere mode we also show its hybridization weight $H^{\perp|\mathrm{TM}}_{e17nl}$ ($H^{\perp|\mathrm{TE}}_{o17nl}$). The NPs distance is not to scale.

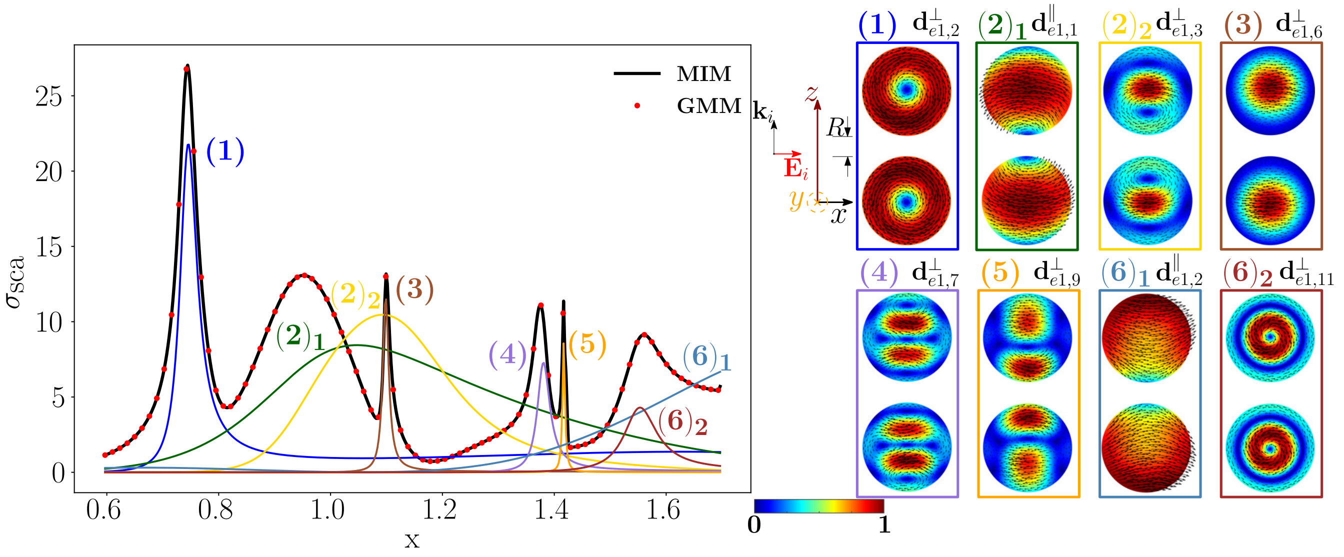

Figure S23. Scattering efficiency σ_{sca} of a Si-spheres homo-dimer as a function of the spheres size parameter $x = 2\pi R/\lambda$, obtained via the material-independent-mode expansion (black line) and by direct-calculation[4] (red dots). The radius of each sphere is R, the edge-edge distance is R. The dimer is excited by a plane wave propagating along the dimer axis and polarized orthogonally to it. Partial scattering cross section (in color) of eight dominant dimer modes whose real part projections on the $y = 0$ plane are shown on the right.

from the second order electric dipole.

The second peak of the σ_{sca} spectrum is a results of the interaction between the dimer-modes $\mathbf{d}^{\parallel}_{e1,1}$ and $\mathbf{d}^{\perp}_{e1,3}$. In particular, the mode $\mathbf{d}^{\parallel}_{e1,1}$ is dominated by the single-sphere fundamental dipole (see Fig. S25).

The third peak of the σ_{sca} spectrum arises from the contribution of the dimer mode $\mathbf{d}^{\perp}_{e1,6}$. As shown in Fig. S26, it results from the hybridization of several isolated-sphere modes. The second order electric dipole $\mathbf{e}^{\mathrm{TM}}_{e112}$ is dominant. Nevertheless, the contributions of the fundamental magnetic quadrupole $\mathbf{e}^{\mathrm{TE}}_{o121}$ and third order electric dipole $\mathbf{e}^{\mathrm{TM}}_{e113}$ are very significant.

References

1. Forestiere, C. & Miano, G. On the nanoparticle resonances in the full-retarded regime. *J. Opt.* **19**, 075601 (2017).
2. Palik, E. D. *Handbook of optical constants of solids*, vol. 3 (Academic press, 1998).

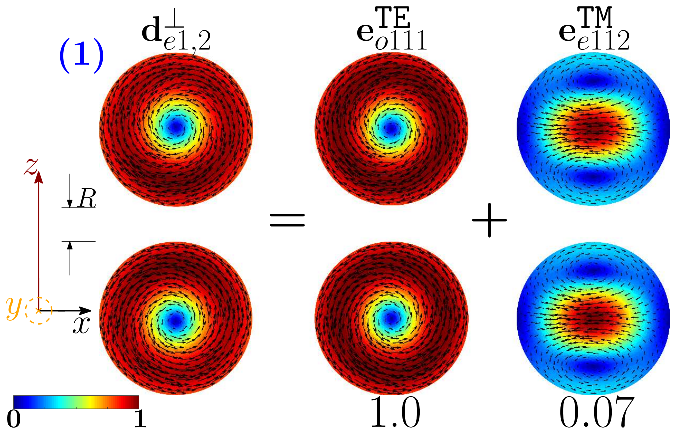

Figure S24. Decomposition of the dimer mode $\mathbf{d}^{\perp}_{e1,2}$ at $x = 0.744$ in terms of hybridizing isolated-sphere modes (real part of the projection on the $y = 0$ plane). Each isolated sphere modes is multiplied by the expansion coefficients of Eq. 8. Below each isolated-sphere mode we also show its hybridization weight $H^{\perp|\mathrm{TM}}_{e12nl}$ ($H^{\perp|\mathrm{TE}}_{o12nl}$). The NPs distance is not to scale. The NPs distance is not to scale.

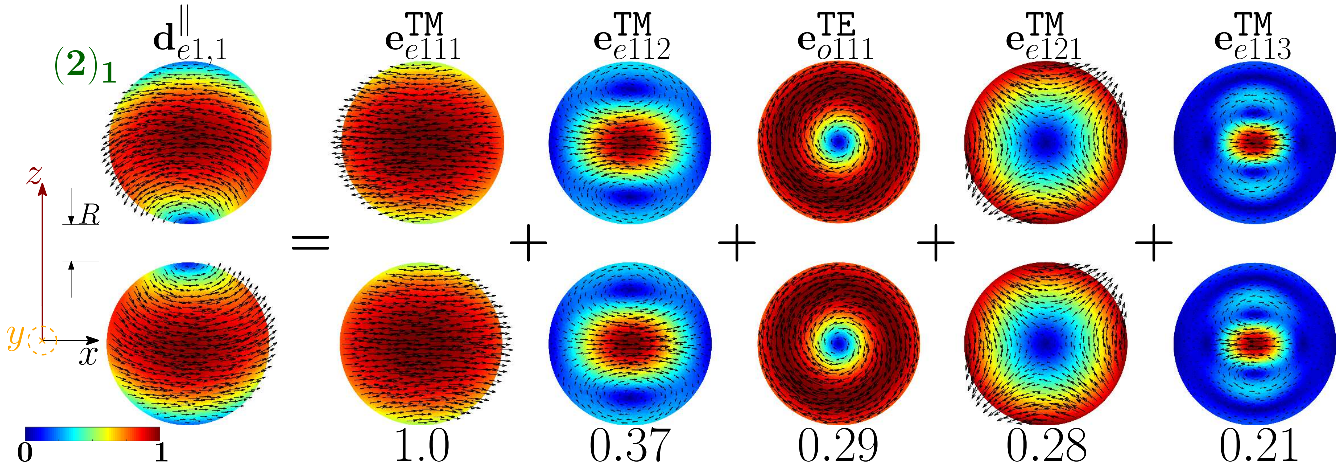

Figure S25. Decomposition of the dimer mode $\mathbf{d}^{\|}_{e1,1}$ at $x = 0.952$ in terms of hybridizing isolated-sphere modes (real part of the projection on the $y = 0$ plane). Each isolated sphere modes is multiplied by the expansion coefficients of Eq. 8. Below each isolated-sphere mode we also show its hybridization weight $H^{\||\mathrm{TM}}_{e11nl}$ ($H^{\||\mathrm{TE}}_{o11nl}$). The NPs distance is not to scale.

3. Mayergoyz, I. D., Fredkin, D. R. & Zhang, Z. Electrostatic (plasmon) resonances in nanoparticles. *Phys. Rev. B* **72**, 155412 (2005).

4. lin Xu, Y. Electromagnetic scattering by an aggregate of spheres. *Appl. Opt.* **34**, 4573–4588, DOI: 10.1364/AO.34.004573 (1995).

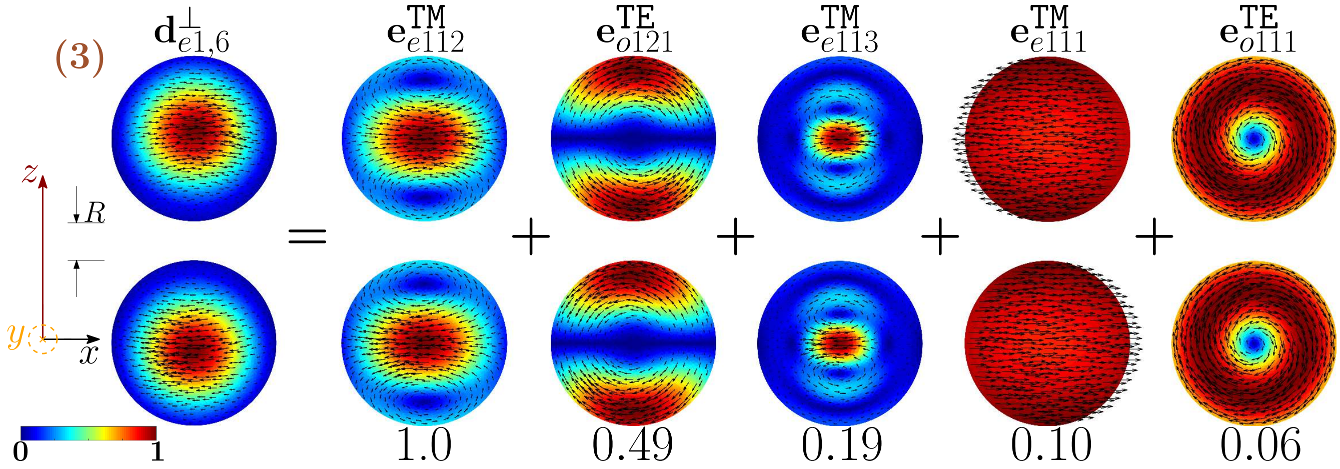

Figure S26. Decomposition of the dimer mode $\mathbf{d}^{\perp}_{e1,6}$ at $x = 1.1$ in terms of hybridizing isolated-sphere modes (real part of the projection on the $y = 0$ plane). Each isolated sphere modes is multiplied by the expansion coefficients of Eq. 8. Below each isolated-sphere mode we also show its hybridization weight $H^{\perp|\mathrm{TM}}_{e16nl}$ ($H^{\perp|\mathrm{TE}}_{o16nl}$). The NPs distance is not to scale.
